# A qualitative evaluation of New Zealand consumers perceptions of general practice nurses

**DOI:** 10.1186/1471-2296-14-26

**Published:** 2013-02-22

**Authors:** Elizabeth J Halcomb, Kath Peters, Deborah Davies

**Affiliations:** 1 School of Nursing and Midwifery, University of Western Sydney, Penrith, Australia; 2 Clinical Nurse Specialist (Lead) Primary Health Care, Health Care Development, MidCentral District Health Board, Palmerston North, New Zealand

**Keywords:** Primary health care, Patient satisfaction, Practice Nursing, Qualitative research

## Abstract

**Background:**

An important consideration in health service delivery is ensuring that services meet consumer needs and that consumers are satisfied with service delivery. Patient satisfaction can impact on compliance with suggested treatments and therefore impact on health outcomes. Comparatively few studies have explored consumer satisfaction with nurses in general practice.

**Methods:**

A sub-group of 18 consumers from a larger quantitative evaluation of consumer satisfaction with New Zealand general practice nurses participated in semi-structured telephone interviews. Interview data was analysed using thematic analysis.

**Results:**

Four major themes emerged from the data. These themes highlighted that, despite confusion experienced by some consumers regarding the practice nurse role, consumers were happy with the level of care provided by them. Consumers felt valued by Practice Nurses and considered them competent and highly knowledgeable. Findings also convey that consumers appreciate the accessibility and financial benefits of utilising the services of practice nurses.

**Conclusions:**

Consumers are highly satisfied with practice nurse service delivery and value their relationships with these health professionals. Consumers revealed that greater clarity around the practice nurse role and their scope of practice may enhance their utilisation. Spreading the message of practice nurses being the right person to deliver care, within their scope of practice, at the right time may have the potential to provide more timely care within the primary care setting.

## Background

The growing burden of chronic and complex disease, and population aging have driven a significant growth in primary health care services internationally [1]. In New Zealand some 80% of the population will visit their general practice annually [2]. To meet this growing demand there has been significant evolution of the primary health care workforce. In particular, there has been substantial growth and development in nursing services provided within general practice [3]. Indeed, many patients will visit their general practice and see only the practice nurse to receive health care services [4].

Given the rapidly changing nature of the primary care workforce it is important that the views of the community and the recipients of the growing general practice nursing services are explored. The importance of involving the community in health service development and evaluation has long been recognised [5]. Indeed, patient satisfaction has been identified by the World Health Organisation as one of the five criteria for good health care quality [6]. As higher levels of patient satisfaction have been linked to improved compliance with therapeutic regimes and better clinical outcomes it behoves researchers and policy makers to explore these issues in a timely manner [7,8].

Despite the growing attention paid to satisfaction with general practice, internationally, limited research has been undertaken to explore consumer perceptions of general practice nursing [9-12]. Whilst some general practice satisfaction scales include a small number of items about nursing services, few collect sufficient information to provide a detailed insight into consumer perceptions of general practice nurses [12]. With the shifting focus to team-based models of care in general practice, it is timely to increase the focus on consumers’ perceptions of nurses as health professionals in their own right.

A small number of Australian studies have identified broad consumer support for the general practice nurse role [13,14]. A limitation of these investigations, however, is that many participants had not actually received services from a practice nurse, but rather were providing their opinion about how they thought they would feel about receiving services from a nurse in that setting.

A number of studies have investigated patient satisfaction with nurse-led clinic interventions [15,16]. In their Australian paper, Mahomed et al. [15] reported the development of a grounded theory of patients’ satisfaction from a larger study exploring a practice nurse-led model of chronic disease management. This study described how patients undergo a cyclical process of what they described as “navigating care”. This cycle incorporated the three stages of determining care needs, forming relationships and having confidence [15]. Failure to adequately develop any of these stages increased the chance that the participants would opt out of the nurse-led care intervention.

In New Zealand, Marshall et al. [16] reported data from an 8-item survey on consultation satisfaction related to healthy lifestyle clinics. These items focussed broadly on satisfaction with the consultation, exploring factors such as adequacy of the consultation room and impact of the education provided on confidence and health. Despite a relatively low response rate, respondents provided over 85% agreement with all items, except the ability to book an appointment easily. This highlights the challenge of the ceiling effect found in many quantitative measures of patient satisfaction [17].

Given that these studies focus on patients who are participating in a trial of nurse-led care, participants may not reflect the broader general practice population. It is timely, to explore consumers’ experiences of receiving services from general practice nurses in the naturalistic setting.

## Methods

### Sampling and recruitment

Ninety-one practice nurses from 20 general practices in New Zealand participated in a study to evaluate consumer satisfaction with general practice nurses. 1505 consumers completed a survey about their levels of satisfaction. These survey data have been reported elsewhere. All survey participants were asked to provide their contact details to the research team if they were interested in participating in follow-up interviews. In total 34 individuals provided their contact details to the research team. The research assistant contacted these individuals via telephone randomly to confirm their consent to participate and arrange a mutually convenient time for an interview. Individuals who were not able to be contacted on the first attempt went to the bottom of the list and further attempts to contact were made if other participants were not recruited.

Informed consent was provided by each consumer before data were collected. Interviews were conducted via telephone and used a semi-structured interview guide to allow the interviewer to probe into issues that arose (Table 1). These questions were drawn from a review of the literature, the project teams’ previous experience and the preliminary survey findings [12]. Interviews were digitally recorded and transcribed by an independent transcription company. Data collection continued until data saturation was achieved, that is, that no new data emerged from subsequent interviews. Saturation was achieved after 18 interviews.

**Table 1:** Question route


1. Thinking about the time when you receive health services from the practice nurse:
a) What was the nature of this care that you received from the practice nurse (prompt – vaccination, managing wounds, counseling, lifestyle advice, checking medications, ECG, taking blood)?
b) How did you come to see the practice nurse for these services?
c) How often do you see the practice nurse?
2. What do you think about the care you get from the practice nurse?
a) What does the nurse do well (prompt – willing and able to answer your questions, show respect and concern, treat your problem)?
b) What could the nurse do better? How could the nurse improve the care that they provide?
3. If you had a choice would you continue to receive these services from the practice nurse in the future? Why/Why not?
4. What do you see as the GPs role in your practice?
5. What do you see as the practice nurses’ role in the practice?
6. What do you think the nurses’ should do more of?
7. What do you think the nurses’ should do less of? Any other comments?

### Ethical considerations

This study was approved by the Human Ethics Committee of the University of Western Sydney. To preserve anonymity, all participants mentioned in this paper and any reports stemming from the data have been allocated a pseudonym. Where names of health professionals have been provided by the participants these have also been allocated a pseudonym in the reports and publications.

### Data analysis

Thematic analysis was undertaken by two researchers in order to ensure findings portrayed a collective representation of participants. Using the process described by Braun & Clarke [18], narratives in participants’ transcripts that represented similar experiences were grouped into codes and then categorised into themes. These themes were discussed at length until consensus was reached that the interpretation of the data was an accurate interpretation of participants’ spoken word.

### Rigour

The principles of credibility and confirmability were used to demonstrate rigour in this study. The involvement of two researchers in the process of independently analysing the interview transcripts and developing themes provides credibility [19]. Confirmability is demonstrated via the use of verbatim quotes to provide the participants voice, rather than purely the researchers interpretations of the data [19].

## Results

Of the 18 consumers who participated, there were 13 women (72.2%) and 5 men (27.8%). Participants were from across the adult lifespan. From the information disclosed, approximately half of the participants visited the practice nurse for issues related to a chronic health condition. Three participants (16.7%) had regularly attended the practice nurse for assistance with weight management. Four of the female participants (22.2%) described attending the practice with their children. The interview data revealed four themes. These themes and their sub-themes are described in Table 2.

**Table 2:** Themes & sub-themes

**Theme**	**Sub-themes**
Satisfaction with practice nurses: “they are first class”	a) Being valued: “She listened to me and treated me like a person”
	b) Developing relationships: “You build up a rapport with them”
Confidence with practice nurses: “she knows what she’s doing”	a) Appreciating practice nurses’ knowledge: “They explain things and that sort of puts me at ease”
	b) Appropriate referral : “I know she’d get the doctor”
Confusion with the practice nurse role: “when you say a practice nurse, I’m a little bit unsure what you mean”	
Appreciation of Practice Nurse accessibility: “It makes it a lot easier for me”	a) Being readily available "I don't have to wait around"
	b) Affordable care: So easy on my funds

### Satisfaction with practice nurses: “they are first class”

Overall, participants were extremely happy with the care delivered by Practice Nurses and this is illustrated in the following excerpts from their transcripts: “*I just think they’re* [PNs] *marvellous*” (Melanie), “*they are first class*” (James), “*She* [PN] *is the best thing that ever happened in my life*” (Chrissy). This was characterised by two sub-themes; the way in which the PNs valued the consumers and listened to them and the development of ongoing rapport between consumers.

a) Being valued: “She listened to me and treated me like a person”

Several participants described how the practice nurses “*listened to me and she treated me like a person”* (Chrissy). Another participant conveyed “*They didn't talk down to me because of my nationality which is good because some doctors take one look at me and then they talk so slow that I did start to get a bit angry”* (Jacqui).

In contrast to their experiences with doctors, some participants expressed how they felt valued by Practice Nurses

*I see the doctors as in a hurry and they get to the point of it and they make you feel - if it's something more serious - they make you feel like you've got to the cause of the problem. Whereas nurses, in my experience, just being that little bit more slower, calmer and they're a bit more personalised. They talk to you; they know your children, especially if you've been going somewhere for a long time like I have - I've just been very lucky* (Natalie)*.*

b) Developing relationships: You build up a rapport with them

Participants also described the benefits of developing an ongoing relationship with the practice nurse. “*You build up a rapport with them and you feel confident with them”* (Millicent). “*They don’t sort of abandon you after the visit. You go back, they contact you”* (Melanie).

The willingness of practice nurses to establish and build a rapport with the consumer and their family was highly valued. “*They always remember who you are. They remember the children’s name which I always think’s great”* (Natalie).

In contrast to the benefits of an ongoing relationship, some participants described the negative impact of not having an established relationship with the practice nurse;

*I had somebody that filled in for someone. I’m really quite a private person. I really find it quite hard going to, you know - I … know that nurse and that’s who I go to. She gets to know me* (Eliza).

### Confidence with practice nurses: “she knows what she’s doing”

a) Appreciating practice nurses’ knowledge: “They explain things and that sort of puts me at ease”

Practice nurses were perceived by consumers as being knowledgeable and competent.

*in the fact that her manner, her speaking and also her professionalism, the way she goes about her job, gives the customer, the patient, the confidence that, hey, she knows what she’s doing. I feel comfortable about it* (James).

Consumers very much appreciated the detailed explanations given by Practice Nurses about their chronic conditions, the need for particular treatments or lifestyle changes.

*They [Practice Nurses] explain things. They actually explain why they're doing what they're doing and that sort of thing so it puts me at ease. Even though I've had them [tests] all before but it's a different way of them explaining it all to me. I could understand it* (Jacqui)*.*

*And often when I haven’t been able to take much pain relief, and they’re very good at explaining why.... I mean the doctors just haven’t got time have they? Well that’s how I feel anyhow. The doctor’s good too and often he’s not there so I would say they are very good at that sort of thing* (Melanie)*.*

*Oh she's - how would you describe her - she's - she tells you what you need to know directly but indirectly. She's able to - she's tactful in how she tells you. There's some things you don't want to hear - sort of stuff - or you know but she just confirms it and that. I think I [unclear] want to know and I have learnt things that I need to be doing and that is all about, sometimes, you've got to pull your head in and do that* (Violet)*.*

b) Appropriate referral: “I know she’d get the doctor”

Underpinning this was the consumers’ confidence that “*as soon as they see something’s not quite right, the doctor comes in*” (Melanie). Whilst there was some indication that consumers wanted to retain the choice to see the GP if they chose to, the confidence that the PN would seek the advice of the GP was seen as comforting.

*I would probably go to the doctor first if I could get in, which is a little bit of an issue up at this clinic at the moment, I’d probably go to the doctor, but having said that, if there was an issue I’d be perfectly happy to go to the practice nurse because I know she’d get the doctor* (Melanie)*.*

*If I had a really serious incident, as you would call it, I would prefer a doctor to be available rather than a nurse.... I’m assuming, incorrectly or what, that a doctor would have a little bit more in-depth knowledge of determining or diagnosing what’s wrong than a nurse* (James)*.*

Participants spoke positively of the PN role in triaging when they needed medical attention.

*She’s my first port of call and if I feel I have an emergency which is not a hospital emergency, then I will talk to a practice nurse* (Ben)*.*

*The other time when I see my practice nurse is when I need something urgently. Now I’ve had occasion when I’ve had diarrhoea, that type of thing, or when I’ve felt really crook and I speak first of all to a practice nurse who then decides whether, well, usually I have to admit, usually decides then I’ll see a doctor* (Ben)*.*

### Confusion around the practice nurse role: “*when you say a practice nurse, I’m a little bit unsure what you mean*”

For a couple of participants it was unclear who was a PN within their practice or what a PN is. *“Well even the nurse that gave me the injection, …she was wonderful and she, I suppose she’s a practice nurse too really isn’t she”* (Miriam). In referring to pathology blood collectors one participant commented. *“Whether you would call those practice nurses or not I don’t know*. (Ben)”.

A few participants described how the role of the PN had changed. “*I think years ago they were just there. They came in when you were with the doctor and stood while you had a smear or whatever, but now they’re far more up front with what they do”* (Melanie). Melanie went on to explain *“I’m lucky I’ve been round the health system for yonks, and I wonder like sometimes if people ring up and make an appointment and there’s no appointments, do they understand that the practice nurses are available. Maybe they could be a little bit more prominent*”.

Several participants did indeed express a lack of clarity about the PN role and the scope of their practice.

*That’s where it’s quite grey to me because I don’t think it’s ever been made clear all the things that you could use the nurse for. So my usual port of call is usually the GP. It’s just been like the vaccinations and smear tests that I’ve ended up seeing the nurse instead* (Sharon).

*When you say a practice nurse, I’m a little bit unsure what you mean because, you see, now there is a nurse there that does nurse-type things for the doctor, you know, like bandages and looking after your wound, stuff like that…Then there’s the nurse … went through your notes, checked on your medication and make sure you’re doing it right* (Barry).

Participants tended to base their perception of the PNs scope of practice around their experience of nursing services. “*Yeah, definitely the vaccinations and smear tests I go to the nurse. There’s been a few things. Like my daughter sprained her ankle really bad a year ago. I ended up seeing the GP. I mean, if I could have gone to the nurse I would have done that if I’d known I could* (Sharon)”.

Once participants had gained an appreciation of the practice nurse and their role they indicated that they returned to the nurse. *“I’ve probably only really started to see them in the last two to three years because up until then I wasn’t really aware that they were there and available to me* (Sharon)”.

A key PN role identified by consumers was that of substitution for the GP in simple tasks. Having the PN take on these tasks was seen as time saving for GPs, particularly in a context of workforce shortage. “*There are things, those superficial things that it’s much better to go to a practice nurse because it’s not wasting his time”* (Melanie).

*I’m very happy for them to stitch me up or tape up whatever’s happening any of those sorts of things – anybody who’s trained can do those sorts of jobs. I’m not saying the jobs are too easy, but a nurse can strap up a sprained wrist with as much skill as a doctor can* (Michelle)*.*

### Appreciation of Practice Nurse accessibility

a) Being readily available: "I don't have to wait around"

A common complaint among participants was the difficulty that they encountered in making appointments and receiving treatment from a GP.

*Once upon a time we could just make an appointment there and then get in today and see our doctor. It’s not like that anymore. You’ve got to make appointment perhaps for – make an appointment today for next week perhaps or even, you know, two or three days’ time when they’re on duty* (Miriam)*.*

Contrary to this experience of having to wait for extended periods for a GP appointment, Practice Nurses were seen as being readily available for consultations. Consumers valued not having to wait to see the Practice Nurses and appreciated them being contactable by telephone if support or advice is required.

*I’m waiting for this hip replacement, they’ve been marvellous. When things have gone wrong, I’ve been able to see them straight away* (Melanie)*.*

*I don’t normally – I certainly don’t to have to wait round very much to get to see a practice nurse and if I do, I simply leave a message and they’ll call me back, probably within a half hour, but certainly within the hour. It is what I’d call virtually immediate access* (Ben)*.*

*It’s usually pretty good. It can be a lot quicker than waiting to see the doctor. You can guarantee if you want to see a nurse you can get in today* (Sharon)*.*

b) Affordable care: “So easy on my funds”

Accessibility of practice nurses was also enhanced due to the lower financial cost for consumers. This was particularly important for consumers who were low income earners and pensioners.

*Yeah, because - and not only that - I suppose it actually - it takes the pressure of the doctors for, you know, some of the things. Not only that, it makes it a lot easier for me. It doesn’t become so costly because, you know, some of the things I see the practice nurse for it doesn’t sort of tie up the doctor really, you know, makes it so easy on my funds too* (Bill)*.*

*We pay $20 when we go to see the care nurse but we pay about $39.90, I think it is, to see the doctor. That for us really is very helpful* (Abbey)*.*

Participants also reflected on the value for money in seeing the GP rather than the PN. *“Sometimes I think god, that was a useless visit. I paid $51 for nothing. I could have got that off* [the PN]” (Violet).

## Discussion

Similar to previous literature that explored consumer satisfaction with PN performance [12], participants of this study regarded the care delivered to them by PNs as positively contributing to their health care experiences. Such high consumer satisfaction with PNs may assist in increasing patients’ compliance with recommended treatment and health promotion strategies [7,8]. This finding provides further evidence to support the importance of the PN role in health promotion and chronic disease management.

Contrary to concerns highlighted by participants in previous research that PNs may act as gatekeepers and impede consumer access to the GP [20], participants in this study expressed confidence that PNs would refer them to the GP if their condition warranted it. However, despite considering the PNs level of knowledge to be exceptional and trusting them to appropriately refer, consumers still expressed a preference to see the GP if they choose. This resonates with findings from existing literature which highlights that although participants perceived PNs showed more compassion and allocated more time to their consultations, the GPs opinion was more highly valued and therefore appointments with them favoured [10,11,21].

Participants’ preference to see their GP instead of a PN may stem from a lack of understanding related to the role and scope of practice of the nurse. Indeed, consumers in this study expressed confusion about what tasks the PNs could do. This is consistent with the literature which describes a level of confusion amongst New Zealand consumers about the PNs scope of practice and clinical skills [22-24]. Similarly, GPs have expressed confusion and uncertainty regarding the scope and expectations of the PN role [3,25]. Reasons for this uncertainty may be in part blamed on the disparate qualifications and variable skill mix of nurses employed in general practice. General practices may employ a mix of enrolled, registered and advanced practice nurses, as well as nurse practitioners under the Practice Nurse title [26]. These varying qualifications not only impact on the PNs scope of practice but also the level of further education that they can undertake to increase their skill base.

With the introduction of New Zealand’s Care-Plus model that promotes a team-based model of care, understanding the roles and scope of practice of various health professionals is becoming increasingly important [22]. To facilitate consumers effectively navigating the maze of team-based care it is important that they have a clear understanding of which health professional to consult for what issues. Given the continuing evolution of primary care to meet the growing demands of chronic and complex disease, there is a need for ongoing dialogue between health professionals and consumers about the various scopes of practice and changing role boundaries as tasks are delegated and health professionals substituted.

### Limitations

Qualitative interviews are useful to provide insight to the experience of individuals where little is previously known. These interviews provided a new insight into the experience of general practice consumers across New Zealand following consultation with a practice nurse. One of the major limitations of this study is that the convenience sample who participated in this study are not necessarily representative of the wider population of general practice consumers. The design of the study did not allow us to identify demographic differences between those who agreed to participate and those who declined to be involved.

Telephone interviews were used to collect data in this study as a means of overcoming the geographical distances between participants and the interviewer. Additionally, this strategy allowed data to be collected at a time convenient to the participant. Whilst use of the telephone provided participants with a degree of anonymity, the separation between the participant and the interviewer may have hindered the development of rapport [27].

## Conclusions

In a country where practice nursing has been well established for many years, we sought qualitative responses from consumers regarding their satisfaction with service delivery by practice nurses. We confirmed findings of previous studies in which patients highly valued the role of the PN but wanted to retain the choice to see a GP when they felt it was necessary.

The findings of this study suggest that, if exposed to practice nurse led care, patients view this positively and continue to seek it out. However, we demonstrated that, although PNs have been an important part of primary care for many years, there is a need for better communication with consumers about the roles and scope of practice of PNs. The findings from this study may lead to further investigations about the acceptability and satisfaction with specific PN interventions.

## Competing interests

The authors declare that they have no competing interests.

## Authors’ contributions

DD and EH were responsible for the study conception and design. EH oversaw the data collection. KP and EH performed the qualitative analysis. EH and KP were responsible for drafting of the manuscript. DD made critical revisions to the paper for important intellectual content. All authors read and approved the final manuscript.

## Pre-publication history

The pre-publication history for this paper can be accessed here:

http://www.biomedcentral.com/1471-2296/14/26/prepub
